# From Pixels to Predictions: Integrating Machine Learning and Digital Image Correlation for Damage Identification in Engineering Materials

**DOI:** 10.3390/ma19010077

**Published:** 2025-12-24

**Authors:** Mostafa Sadeghian, Arvydas Palevicius, Jokubas Sablinskas, Paulius Griskevicius

**Affiliations:** Faculty of Mechanical Engineering and Design, Kaunas University of Technology, Studentu 56, 51424 Kaunas, Lithuania

**Keywords:** machine learning, digital image correlation, damage identification

## Abstract

Damage assessment in engineering materials is essential for structural reliability and safety. While traditional imaging techniques and Digital Image Correlation (DIC) provide valuable insights into deformation and crack evolution, they often require significant manual effort and suffer from accuracy limitations under complex loading conditions. Recent advances in Artificial Intelligence (AI), particularly Machine Learning (ML) and Deep Learning (DL), have enabled the development of automated, high-resolution, and near real-time damage assessment techniques. This paper reviews methods that integrate ML with DIC to assess damage in composites, metals, and other engineering materials. We compare conventional ML models with modern DL architectures, discuss key challenges, and propose future research directions. The findings demonstrate that coupling DIC with ML significantly improves the accuracy, speed, and reliability of damage identification in engineering materials.

## 1. Introduction

Recently, Artificial Intelligence (AI) has become widely recognized as a powerful technology that can change many industries around the world. Its various applications demonstrate a significant impact across scientific and engineering fields [1,2]. The term “AI” was first introduced by John McCarthy in 1955 [3]. AI is defined as a domain within computer science that aims to simulate human cognitive functions (such as learning, reasoning, and adapting) through computational systems [4]. It enables machines to perform complex tasks by copying human problem-solving processes. AI uses different methods, including Machine Learning (ML), Deep Learning (DL), and rule-based programming. These approaches allow systems to operate with a degree of autonomy and intelligence. ML involves training models on extensive datasets to detect patterns and make predictions or decisions without being explicitly programmed. DL is a part of ML that uses layered neural networks to process complicated and unorganized data. In contrast, rule-based systems function through predefined logic and structured rules that guide decision-making processes. By combining these different methods, AI systems can effectively address complex, multi-dimensional problems. ML enables systems to learn from historical data and build predictive models without explicit programming [5,6,7].

Crack identification using image processing is generally divided into conventional methods and ML-based techniques. In traditional image processing, the procedure usually starts with acquiring the image and applying preprocessing operations such as filtering or morphological adjustments. After that, feature information is extracted through approaches such as edge detection and threshold segmentation, where the accuracy of this step plays an important role in the final identification results. The process is then followed by post-processing to determine the location, shape, orientation, and size of the cracks.

Recently, Digital Image Correlation (DIC) has been widely used to analyze how materials deform under stress [8,9]. It does this by comparing images taken before and after a load is applied, giving full-field quantitative insight than traditional image processing methods. The technique works by evaluating the degree of similarity between images, often through the application of random speckle patterns spread across the surface of the specimen. The method uses images to calculate the displacement field of sample points, which helps in detecting deformation and cracks [10]. Tong [11] examined the robustness, reliability, and computational efficiency of four correlation criteria by employing three distinct image sets that varied in brightness, contrast, local illumination, and blur. Similarly, Gehri et al. [12] presented an automated DIC-based study for crack detection and quantification in structural materials. Their approach successfully identified and measured cracks through displacement field analysis. This capability significantly reduced the amount of manual work needed. In a related study, Panwitt et al. [13] introduced an automated approach to calculate crack lengths in mixed-mode fatigue tests through DIC. Their method proved to be highly accurate in tracing crack growth even under complex loading conditions. The use of DIC has since extended to many areas of solid mechanics [14], ranging from experimental mechanics [10], fracture mechanics [15], and fatigue studies [16] to mechanical testing [17]. It has also been applied to a wide variety of materials, including metals [18], concrete [19], and composites [20].

Nevertheless, conventional image-based approaches, including DIC, still struggle with issues of accuracy and the continuous monitoring of crack development. In particular, DIC measurements are affected by speckle quality, lighting variations, out-of-plane motion, and noise amplification during strain differentiation, which limit their independent reliability for automated damage detection. To address these shortcomings, ML methods have gained considerable attention in recent years for crack detection tasks [21,22,23,24]. Different convolutional neural network (CNN) architectures such as U-Net, LinkNet, Feature Pyramid Network, and DeepLabv3 have been applied, often in combination with traditional image processing strategies such as the Otsu thresholding technique, to improve the identification of cracks. Pham et al. [25] presented a method that combines DL with image processing to automatically detect and measure the growth of surface cracks. Also, Hongwei Hu et al. [26] developed an enhanced approach for road crack detection by adapting the YOLOv5 model to analyze images captured from vehicle-mounted cameras.

In addition, a recent study introduced a comprehensive approach that combines an improved YOLOv7 model, a crack growth assessment technique, the enhanced DeepLabv3+ model, and image processing tools to track the full progression of cracks. This work enables both near real-time under controlled experimental conditions and continuous monitoring of crack growth dynamics [27].

ML techniques are typically grouped into four major categories: supervised learning, unsupervised learning, semi-supervised learning, and reinforcement learning [28]. Supervised learning involves training an algorithm on labeled data, enabling it to make predictions for new, unseen examples. This approach is widely used for both classification and regression tasks, especially in the prediction of material properties. In contrast, unsupervised learning focuses on uncovering hidden patterns or structures in data that lack labeled outputs. Semi-supervised learning serves as a hybrid approach, combining small amounts of labeled data with larger volumes of unlabeled data, with a stronger emphasis on the latter during training [29,30].

Reinforcement learning, on the other hand, is a distinct form of ML in which algorithms interact with and learn from dynamic environments by trial and error, refining their actions based on feedback to improve future outcomes [31].

DL is one type of ML that utilizes artificial neural networks (ANNs) with more than two hidden layers. These multilayered architectures allow DL models to automatically find useful features in raw data, which removes the need for manual feature selection. With layer-by-layer learning, these models can discover complex patterns and abstract ideas from data, which makes them very useful for tasks such as speech recognition, natural language processing, and others [32,33,34,35]. Several DL techniques have proven effective in predicting material properties, including CNNs, deep belief networks (DBNs), recurrent neural networks (RNNs), and generative adversarial networks (GANs).

**Figure 1:**
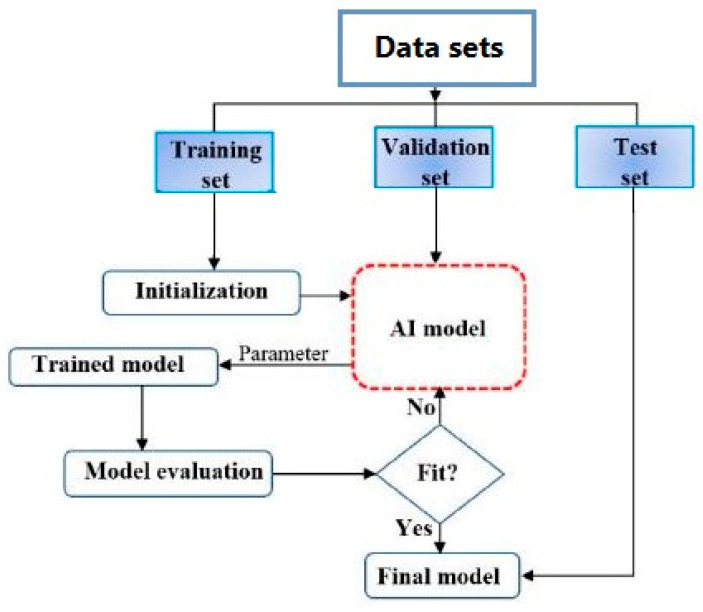
General procedure used in AI-driven prediction approaches.

In AI applications for predicting material properties, raw data is commonly split into three distinct subsets: the training set, the validation set, and the test set. The training set is used to train the AI model by enabling it to identify underlying patterns and correlations within the data. As the model trains, its performance is monitored using the validation set, which helps to detect overfitting (an issue where the model fits the training data too closely and struggles to make accurate predictions on new data). This validation process also helps adjust the model’s settings that control how it learns. Once the model is both trained and optimized, its ability to make accurate predictions is evaluated using the test set, a separate dataset that provides an unbiased measure of the model’s generalization performance on new, unseen data. This stage is necessary for evaluating how well the model generalizes to new data and offers an estimate of its effectiveness in practical, real-world applications. Figure 1 illustrates the standard workflow of AI-driven prediction approaches.

Despite significant advances in both DIC and ML-based damage detection, current studies still suffer from several critical limitations, including the lack of standardized benchmark datasets, limited generalization under varying experimental conditions, insufficient integration of physical constraints into learning models, and the absence of uncertainty quantification in DIC-driven ML predictions. Furthermore, most existing reviews focus either on ML algorithms or on experimental DIC techniques separately. This review explicitly addresses these gaps by providing a physics-informed comparative analysis of DIC-integrated ML and DL frameworks for automated damage assessment.

## 2. Literature Review ML-Based Structural Damage Assessment

Damage analysis is one of the most important aspects of engineering science, as it plays a critical role in ensuring the safety, reliability, and service life of materials and structures. An accurate understanding of damage initiation, evolution, and failure mechanisms is essential for the design of durable engineering systems and for the prevention of catastrophic structural failures [36,37,38,39,40]. In recent years, the rapid growth of ML has transformed the field of damage analysis, providing accurate, robust, and scalable methods for detecting, localizing, and predicting the severity of damage in various structural and material systems. By using frequency analysis, vibration data, acoustic emission, imaging, and hybrid physics-based approaches, ML overcomes many of the limitations of traditional physics-based and experimental techniques under data-rich and well-controlled conditions, which are often costly, time-consuming, or unreliable under complex loading and environmental conditions [41,42,43].

Frequency- and vibration-based studies have played a significant role in ML-based damage assessment. Vu et al. [44,45], Seo and Han [46], Dugalam and Prakash [47], Lee et al. [48], Hakim et al. [49], and Khatir et al. [50] utilized natural frequencies, frequency response functions, and finite element data combined with ANN, CNN, LSTM, and hybrid ensembles for crack detection and parameter estimation. Similarly, Bokaeian et al. [51], Hassaine et al. [52], Shafighfard et al. [53], Khiem et al. [54], Khalkar et al. [55], Zhan et al. [56], Siow et al. [57], Le et al. [58], Rodrigues de Sousa et al. [59], Avarzamani et al. [60], Nguyen et al. [61], Katı et al. [62], and Pithalis et al. [63] proposed regression, ensemble, and graph neural network-based models for modal analysis, crack depth estimation, and damage quantification.

Acoustic emission signal analysis has also been widely investigated. Barbosh et al. [64,65], Deepak et al. [66], and Huynh et al. [67] used wavelet transforms, empirical decomposition, and CNN models to process acoustic emission or electromechanical admittance signals, achieving over 90% accuracy in laboratory-scale studies, while large-scale field validation remains limited. Complementary works such as Khan and Kim [68], Kashyap et al. [69], and Nguyen [70] introduced unsupervised, Bayesian, and TinyML-enabled approaches to improve acoustic emission and guided-wave diagnostics under temperature and noise variations. However, acoustic emission-based ML models remain highly sensitive to sensor placement, signal loss, and background noise, which significantly limit their robustness in large-scale and long-term monitoring applications.

Vision-based and image-assisted methods have further advanced SHM capabilities. Tabatabaeian et al. [71], Yamada et al. [72], Moreh et al. [73], Meruane et al. [74], Diaz-Escobar et al. [75], Ye et al. [76], Manujesh and Prajna [77], Shableya et al. [78], and Hake et al. [79] applied CNNs, GANs, DNNs, and segmentation models to detect delamination, impact damage, and cracks from C-scan, UAV, microscopy, and 3D point cloud data. These studies confirmed high robustness, with accuracies exceeding 95% in many cases, mainly under controlled imaging and laboratory conditions. Most reported ML accuracies are obtained within narrow training domains, and the generalization capability of these models under varying material systems, loading modes, and environmental conditions remains an open research challenge.

Recently, novel optimization, hybridization, and scalability strategies have been introduced. Some authors, such as Nguyen [70], Khatir et al. [50] and Pithalis et al. [63] used advanced optimization methods to enhance learning and generalization. Others, including Wang and Cha [80] and Rodrigues de Sousa et al. [59], explored unsupervised or semi-supervised novelty detection to address the lack of labeled data in real-world structures.

Several studies have combined ML with finite element modeling (FEM) for damage-sensitive responses. Gomes and Silva [81] analyzed surface imperfections in composite shells using FEM and ML, while Zhai et al. [82] applied ML to calibrate damage models in mega castings. Gunst et al. [83] coupled FE-CZM with LSTM surrogates to efficiently model porosity effects in ceramic composites, and Freed [84] developed surrogate regression models for fatigue crack growth in aluminum alloys. Zhou et al. [85] extended this approach to cracked rotary blades, verifying improvements in stability through a hybrid deep neural network.

Signal-based approaches have also been widely studied. Feng et al. [86] used generalized energy indices with CNN, MLP, and LSTM for beam damage detection, while Du et al. [87] and Zhang and Wang [88] employed acoustic emission data with DL for composite damage and interface crack identification. Zuo et al. [89] proposed a feature-informed CNN for leak detection in aluminum pipes. Lee et al. [90] developed CNN-FCN models for diagnosing compression-induced cracks with over 96% accuracy. Mi et al. [91] combined infrared thermography with U-Net and LSTM to track fatigue crack growth in titanium alloys.

Fatigue and fracture prediction are important areas of research. Sanchez and Wass [92] modeled laminate fatigue life using neural networks for computational efficiency, while Dong et al. [93] proposed a physic-guided semi-supervised method for fatigue classification. Santos et al. [94] predicted fatigue crack paths under mixed-mode loading using ANN and kNN, reducing computational cost by over 90%. Xu et al. [95] (Crack-Net) and Zhu et al. [96] (physics-informed ML) improved fracture predictions in composites, and Yan et al. [97] showed that U-Net-LSTM models could forecast crack propagation more accurately than conventional methods.

Recent studies have also applied ML to special conditions. Phan et al. [98] used Taguchi-optimized Random Forests to predict post-cracking strength in FRCCs. Raftar et al. [99] applied bagged trees to assess hydrogen embrittlement in pipeline steels. Rathore et al. [100] used deep neural networks to capture fatigue crack growth across stress ratios in brittle steels. Chen et al. [101] developed a hybrid EA model for predicting recurring crack self-healing. Wang et al. [102] built a corrosion fatigue model for aluminum alloys optimized with PSO. Hu et al. [103] applied Gradient Boosting for hydrogen-assisted fatigue crack growth. Also, Ye et al. [104] proposed incremental learning to improve fatigue crack growth predictions near the threshold region. Hooshyar et al. [105] proposed an ML-based methodology for damage detection in steel beams, using dynamic response signals processed through advanced time-frequency functions. Using XGBOOST and MTEN algorithms, they demonstrated superior detection accuracy of XGBOOST in identifying both the location and severity of damage, emphasizing the effectiveness of ensemble ML methods in SHM. Yang et al. [106] introduced a multi-level structural damage identification method based on time-domain response reconstruction and deep reinforcement learning, showing improved localization and quantification of damage in large-scale structures with limited sensor data. Finally, comprehensive reviews such as Zhuang et al. [107] and Wu et al. [108] reviewed DL applications in crack detection.

Mirzaei [109] presented an ML-based framework for predicting mixed-mode fracture load and crack initiation angle using stress, strain or displacement field data. The study aimed to enhance prediction accuracy without relying on complex analytical models. Using numerical simulations of cracked specimens, regression models were trained on localized field data. Strain fields provided the most accurate results. The study was computational but relied on DIC principles, which show potential for future experimental use.

ML and DIC have also been applied in non-damage-related analyses. For example, Zhang et al. [110] utilized DIC and ANNs to investigate the strain hardening behavior of 316L stainless steel under tensile loading, demonstrating that these tools can effectively model material behavior beyond failure or fatigue analysis. Zhao and his colleagues [111] developed a strain-guided Kolmogorov Arnold Network DIC method to improve the accuracy of full-field deformation measurements under small and complex displacements.

Rishad et al. [112] conducted a study to analyze the stress distribution in carbon fiber-reinforced polymer (CFRP) bonded joints, aiming to better understand the mechanical performance of these joints under various configurations and loading conditions. Their results revealed that joint configuration significantly affects stress distribution and failure risk, and that the combination of ML and DIC enables more reliable evaluation and design of CFRP bonded structures.

Despite the large number of published studies, no widely accepted standardized DIC-based damage benchmark yet exists for quantitatively comparing different ML and DL architectures under identical DIC-based damage scenarios.

In summary, ML has emerged as a powerful tool for damage assessment, enhancing predictive accuracy and reducing computational costs, while its robustness strongly depends on dataset quality, physical consistency, and uncertainty control. Although challenges such as noise sensitivity, data scarcity, and model explainability remain, ML-driven approaches have demonstrated great potential for real-time, automated, and scalable damage detection that contributes to safer and more reliable engineering systems and infrastructures.

## 3. DIC for Analyzing Damage

Imaging-based techniques have become essential tools in mechanical and materials engineering for identifying/detecting damage, assessing structural integrity, and ensuring reliability. These methods enable non-destructive evaluation across a wide range of materials and scales, supporting both research and industrial applications. Among the imaging-based methods are those summarized in Table 1.

DIC provides unique advantages compared to radiographic, ultrasonic, and thermographic techniques. Unlike radiography or ultrasonics, which directly visualize internal flaws such as cracks, pores, or delaminations, DIC does not detect damage explicitly. Instead, it measures full-field displacement and strain distributions on the specimen surface, which act as indirect indicators (proxies) of damage evolution rather than direct measurements of material failure. Consequently, subsurface damage mechanisms such as micro-void nucleation, early-stage fatigue microcracking, and internal delamination cannot be directly resolved by surface-based DIC measurements. This capability allows for the identification of early indicators of damage, since local strain concentrations often precede the formation or propagation of cracks. Moreover, DIC is a completely non-contact optical method, requiring no coupling medium and avoiding exposure to ionizing radiation, which makes it safer and easier to deploy in laboratory and in situ structural testing. It can be applied to complex geometries and a wide range of materials, provided that an adequate speckle pattern is available.

There are two primary configurations of DIC: 2D-DIC and 3D-DIC. The former uses a single camera and is suitable for flat specimens with in-plane deformation, while the latter employs two synchronized cameras in a stereo setup to capture both in-plane and out-of-plane deformations, making it more versatile for complex geometries [120]. The choice between these methods depends on the nature of the test, the dimensionality of deformation, and the geometry of the specimen. To successfully conduct a DIC experiment, a series of specialized components are required. These include high-resolution digital cameras, an illumination system to ensure consistent lighting, speckle patterning tools for surface preparation, a stable mounting system, and software capable of both acquiring and analyzing images. Accurate camera calibration is particularly crucial in 3D-DIC to establish the relationship between image coordinates and physical space [121]. The process of using DIC typically involves surface preparation (speckle pattern application), image capture during mechanical loading, image registration and correlation, and finally the computation and visualization of displacement and strain fields. Recent developments have also led to the use of high-speed DIC systems capable of capturing dynamic events and deformation at high strain rates [122]. The principal advantage of DIC lies in its ability to provide full-field displacement and strain data with high spatial resolution, allowing researchers to assess local variations in material response, identify strain concentrations, and validate computational models such as FEM. It is widely used in applications ranging from composite failure analysis and fatigue crack propagation to material characterization and SHM [42,123,124]. Furthermore, DIC can be coupled with other measurement techniques, including infrared thermography and acoustic emission, to provide a more comprehensive picture of material behavior under complex loading conditions. Despite its advantages, the accuracy of DIC is fundamentally limited by several physical and numerical factors, including speckle decorrelation under large strains, sensitivity to rigid-body and out-of-plane motions in 2D-DIC, the subset size-bias trade-off, and systematic errors introduced during numerical strain differentiation. These limitations impose intrinsic bounds on the reliability of DIC-based damage measurements [125].

To provide a clearer overview of how DIC has been employed for damage identification and characterization in engineering materials and structures, a selection of representative studies is summarized in Table 2. These works are categorized according to the adopted definition of damage, the specific purpose of applying DIC, and the main findings reported. The review highlights that DIC has been successfully applied not only for crack initiation and propagation analysis but also for monitoring localized deformations, validating numerical models, and improving damage identification under complex loading and environmental conditions. Overall, the summarized studies emphasize both the versatility and the current challenges of DIC in advancing experimental mechanics and SHM.

DIC is fundamentally a full-field optical measurement technique for displacement and strain estimation rather than a direct crack detection tool. Unlike conventional vision-based inspection methods that rely solely on grayscale contrast for visual crack identification, DIC quantifies the mechanical response of materials by tracking surface deformation and computing displacement and strain fields. From an industrial perspective, this capability is crucial because most structural damages are governed by localized strain concentration, stiffness degradation, and deformation incompatibility rather than by visually detectable cracks alone [156,157].

In practical engineering applications, DIC is widely used for strain measurement, fracture mechanics characterization, fatigue crack growth analysis, and validation of numerical models. Parameters such as displacement continuity, strain localization, crack opening displacement, and stress intensity factors are directly derived from DIC measurements and constitute the primary data sources for quantitative damage assessment. The suitability of DIC for damage assessment strongly depends on both the material system and the dominant damage mechanism. DIC is particularly effective for crack-driven and strain-localization-dominated failure in metals, composites, and concrete, while subsurface-dominated damages or deeply embedded delamination require complementary NDT techniques for reliable detection [20,157].

Despite its advantages, the industrial deployment of DIC is limited by sensitivity to lighting and speckle quality, noise amplification during strain differentiation, out-of-plane motion errors in 2D-DIC, and high data processing demands. In particular, strain fields computed as spatial derivatives of measured displacements are highly sensitive to high-frequency noise, which may obscure early damage signatures. Therefore, DIC should be regarded as a physics-based experimental technique whose combination with ML enables automated, robust, and physically meaningful interpretation of full-field strain and deformation data [157].

The characteristic properties of DIC strain fields naturally align with specific ML architectures. The strong spatial correlation and localized strain gradients around damage zones motivate the use of CNNs for spatial feature extraction. Temporal continuity of strain evolution under cyclic or dynamic loading provides a physical basis for RNNs and LSTM-based models. Furthermore, strain concentration factors, strain energy density, and gradient-based descriptors serve as physically meaningful engineered features for classical ML algorithms such as SVM and Random Forest. This direct correspondence establishes a physics-guided bridge between DIC measurements and data-driven damage modeling.

## 4. Conventional ML Approaches for Damage Identification in Engineering Materials

### 4.1. Support Vector Machine (SVM)

**Figure 2:**
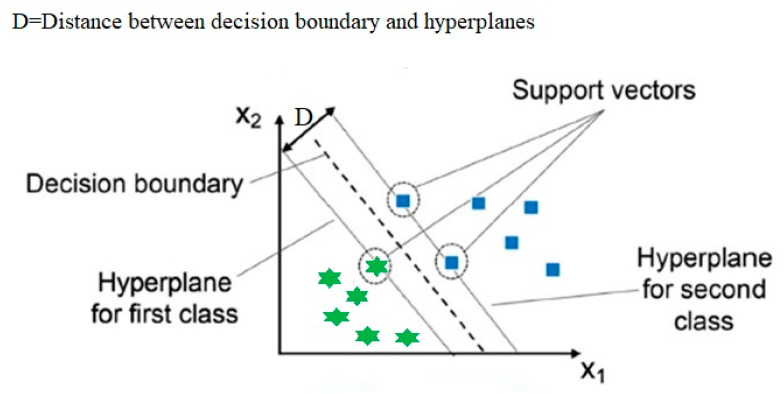
Basic illustration of the SVM approach [158].

SVM, introduced by Cortes and Vapnik in 1995, is a widely adopted supervised learning algorithm designed for classification, regression, and outlier detection. A key principle of SVM is the construction of an optimal decision boundary, known as a hyperplane, which separates data points in the feature space. As illustrated in Figure 2, this hyperplane is determined in such a way that it maximizes the margin (the distance between the hyperplane and the nearest data points from each class, known as support vectors). These support vectors are critical, as they define the position and orientation of the hyperplane. In regression tasks, a variant called Support Vector Regression (SVR) is employed. SVR offers notable advantages, such as computational efficiency that does not scale with the dimensionality of the input space, good generalization performance for low-dimensional, well-engineered DIC features under appropriate kernel selection and regularization, and also high predictive accuracy.

In practical damage identification applications, SVM is particularly effective for small- to medium-sized DIC or vibration datasets with high-dimensional features, where it provides high prediction accuracy and strong generalization. However, its performance strongly depends on proper kernel selection (for example, polynomial kernels) and careful tuning of hyperparameters such as the penalty factor *C* and kernel width. For large-scale experimental datasets, the computational cost and memory requirements of SVM increase significantly, limiting its applicability for real-time damage monitoring. Moreover, SVM is sensitive to noise in experimental measurements, which may degrade classification accuracy if proper feature filtering and normalization are not applied [158].

SVMs have been widely applied for damage identification and durability evaluation in engineering materials, particularly composites. For instance, multi-support vector machines have been employed to classify damage in composite-reinforced natural fibers using image-based features after anisotropic filtering, demonstrating superior accuracy compared to conventional models [159]. In fiber-reinforced composites, nonlinear SVMs with kernel functions have been used to detect damage more effectively by mapping small frequency variations into higher dimensions, improving separation between undamaged and damaged states [160]. Image-based SVM approaches further combine anisotropic diffusion filtering, Fuzzy C-Means clustering, and Zernike moments to extract discriminative features, achieving high accuracy in both global and local damage classification [161]. Moreover, SVM has been applied to composite rotor blades, where vibratory hub loads derived from aeroelastic simulations were used to classify damage stages and predict blade life, showing robustness and practical applicability for prognostics in aerospace structures [162].

### 4.2. k-Nearest Neighbor (k-NN)

The k-NN algorithm is a non-parametric, instance-based ML method used for both classification and regression tasks. Instead of building an explicit model, k-NN stores training data and predicts outcomes based on the distance between a new data point and its k-nearest neighbors in an n-dimensional space. Due to its simplicity, ease of implementation, and minimal need for parameter tuning, k-NN has been widely applied in predicting the mechanical properties of composite materials. However, k-NN also has limitations: its performance degrades with high-dimensional data, it struggles to capture complex feature relationships, and it can be computationally expensive on large datasets. Additionally, selecting an optimal value for k remains a critical challenge that affects prediction accuracy.

Sharma et al. [163], used k-NN to predict the dynamic fracture toughness of glass-filled polymer composites, reducing the need for complex and time-consuming experiments. The model uses geometrical features (such as particle aspect ratio), along with time, dynamic elastic modulus, and volume fraction as inputs. The k-NN algorithm achieved very good accuracy in predicting fracture behavior (by estimating the dynamic fracture toughness and its variation with material design and loading parameters), enabling efficient and accurate toughness estimation.

From an optimization and real-world implementation perspective, the performance of k-NN is highly sensitive to the selection of the neighborhood size (*k*) and the distance metric. Small values of *k* may lead to overfitting, while large values may reduce sensitivity to local damage features [164,165]. In large DIC-based datasets, k-NN becomes computationally expensive during the prediction stage since all training samples must be scanned, making it unsuitable for real-time damage identification. In addition, its prediction accuracy significantly degrades in the presence of noisy strain features unless proper dimensionality reduction and feature scaling are applied.

### 4.3. Decision Tree

Decision tree models are widely used for classification and regression due to their simplicity and interpretability. However, they are prone to overfitting, which can negatively impact their predictive performance. To address this, ensemble learning techniques such as Random Forest were introduced. Random forest regression combines multiple randomly generated decision trees to improve accuracy and robustness. In addition to random forest, boosting algorithms such as AdaBoost and XGBoost have been employed to further enhance prediction accuracy. XGBoost, which uses gradient-boosting optimization, has also proven highly effective, outperforming neural networks and other methods in predicting the strength and ductility of advanced cementitious composites. Overall, decision tree-based models are valued for their interpretability, scalability to large datasets, and relatively low computational cost, though ensemble techniques are often necessary to overcome their inherent limitations [42].

For real-world damage detection, Random Forest and XGBoost models offer improved robustness against experimental noise and feature uncertainty compared to single decision trees. Their optimization typically involves tuning the number of trees, tree depth, and learning rate. While these ensemble methods provide high prediction accuracy and are well-suited for large DIC-based feature sets, their computational cost increases with model complexity, which can limit real-time implementation in high-speed monitoring systems [20,165].

### 4.4. ANNs

In this section, conventional ML refers to algorithms that rely on manually engineered features extracted from DIC displacement or strain fields (in contrast to DL models that perform automatic feature extraction using multi-layer neural networks). Accordingly, conventional ANNs with limited hidden layers and feature-based inputs are considered conventional ML models in this review. ANNs represent one of the most widely used supervised ML techniques, inspired by the structure and function of biological neural networks. A typical ANN architecture is composed of three main layers: an input layer, one or more hidden layers, and an output layer. The neurons in the input layer process input features, while the neurons in the output layer generate predictions. Within the hidden layers, each neuron collects input from the neurons in the preceding layer, integrates this information, and performs a simple computation to produce its output [42].

ANNs are increasingly employed for predicting mechanical properties and damage identification in composites due to their ability to capture complex nonlinear behavior. Studies show their superior performance in estimating stress–strain relations, fracture behavior, and damage evolution compared to conventional ML models. Applications include delamination prediction using FEM data [166], SHM of carbon fiber/epoxy laminates through strain correlations [167], crack detection with laser ultrasonic signals achieving over 99% accuracy [168], and optimized ANN architectures for vibration-based SHM of glass fiber composites, reaching about 100% classification accuracy on limited laboratory datasets. Despite their effectiveness, ANNs require large datasets and careful tuning to avoid overfitting, which is commonly controlled through k-fold cross-validation, regularization strategies, and early stopping, as well as higher computational effort than methods such as SVM or k-NN [169].

In addition to the widely adopted ML techniques discussed earlier, various other methods have been utilized to predict damage in engineering materials, each offering distinct features and advantages. These include linear regression [170], logistic regression [78], fuzzy logic approaches [171], neuro-fuzzy inference systems [172], extreme learning machines [173], and graph neural networks [174,175].

ANN performance depends strongly on the selection of network depth, number of neurons, learning rate, and regularization strategies such as dropout and early stopping. While ANNs can achieve high prediction accuracy for nonlinear damage behavior, their training process is computationally more expensive than that of SVM and k-NN. In real-time damage monitoring, lightweight ANN architectures are preferred to balance accuracy and efficiency. Also, SVM exhibits strong generalization in low-dimensional feature spaces but is highly sensitive to kernel selection and outliers in noisy DIC data. Random Forest provides higher robustness to noise and nonlinear feature interactions but suffers from reduced interpretability and bias under class imbalance. kNN offers conceptual simplicity and fast training but shows poor scalability and strong sensitivity to feature normalization and noise. Conventional ANN models enable nonlinear regression and classification but require careful tuning to avoid overfitting on limited DIC datasets [16,20,164,165].

In classical ML-based DIC damage identification, severe class imbalance between healthy and damaged samples represents a major source of biased learning, since undamaged states typically dominate experimental datasets. This imbalance directly affects SVM decision boundaries, Random Forest voting behavior, and k-NN distance-based classification and often requires cost-sensitive learning, class weighting, or resampling strategies to ensure physically meaningful damage classification.

## 5. DL Methods for Damage Assessment

This section provides a brief overview of the most extensively studied and commonly applied DL techniques for damage assessment in engineering materials.

### 5.1. CNNs

CNNs are widely used in DIC-based damage identification due to their strong ability to extract spatial features directly from full-field strain and displacement data. In damage assessment, CNNs are commonly applied to DIC-derived strain maps, displacement fields, or stress distributions to perform crack detection, damage localization, and pixel-level segmentation. CNNs, originally developed for tasks such as facial recognition and image classification [176], have found broad applications in materials science with the rise in large-scale databases. They are now widely used to predict the mechanical properties and damage behavior of composites. For example, Yang et al. [177] combined principal component analysis with CNNs to predict stress–strain curves beyond elasticity, while other studies applied CNNs to estimate stiffness, strength, toughness, and modulus [178,179]. CNNs have also been integrated with optimization algorithms to improve microstructural design [179].

**Figure 3:**
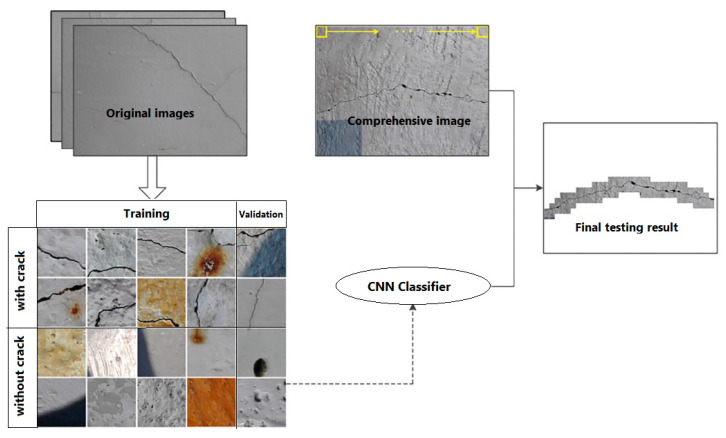
CNN-based procedure for crack detection [185].

Applications extend to damage prediction, such as impact-induced delamination in fiber-reinforced polymers (FRPs) [180], automated porosity segmentation in laminates using X-ray micrographs with U-Net and FCDenseNet [181], and surface crack detection in cementitious composites using CNNs [182]. Advanced models, like ECARNet, further improved composite damage detection accuracy above 98% [183]. CNNs have also been applied in steel-concrete composites for damage localization and severity assessment, showing strong reliability [184]. Overall, CNNs provide accurate, efficient, and automated tools for damage evaluation and material characterization. These studies demonstrate that CNN-based models can achieve high accuracy primarily under controlled laboratory conditions with well-defined loading paths, stable illumination, and high-quality imaging setups. CNN-based models trained on DIC strain fields primarily learn the nonlinear spatial coupling between strain localization patterns, gradient intensification near crack tips, and evolving damage fronts, rather than directly detecting physical material failure. Li and Zhao [185] proposed a method for detecting cracks on concrete surfaces using CNN. Instead of relying on traditional image processing techniques that require hand-crafted feature extraction, the CNN automatically learns crack features from a large database of labeled images. The authors trained and validated their model on 60,000 cropped images and then tested it on high-resolution photos of real concrete structures. Their results confirmed the strong practical applicability of CNNs for large-scale, real-world crack detection with high robustness to surface texture variation and illumination conditions. As can be seen in Figure 3, the authors illustrated the overall workflow of their crack detection method. First, raw images of concrete surfaces are collected and cropped into smaller patches, which are then manually classified as containing cracks or not. These labeled patches form the training and validation datasets. The CNN is trained on this data to learn distinguishing features of cracks and becomes a classifier capable of identifying them automatically. For testing, a new large raw image is scanned using a sliding window, and each patch is classified by the trained CNN. The results of these classifications are combined to generate the final crack detection outcome on the full image.

Ref. [186] introduced a supervised DL framework called StrainNet-LD, specifically designed to estimate large displacement fields from image pairs. The model consists of a CNN with a displacement-field decomposition module that separates rigid-body motion and deformation. It is trained on synthetic datasets containing large and complex deformations to improve robustness. StrainNet-LD takes reference and deformed speckle images as input and directly outputs dense displacement fields, allowing subpixel accuracy even under large strain conditions.

This method significantly improves the reliability of DIC for damage assessment under large deformation and provides high-quality full-field deformation data for subsequent ML-based damage analysis. From a computational efficiency standpoint, CNN-based damage detection models require significant training time and GPU resources, especially for high-resolution DIC strain fields. However, once trained, their inference speed is typically fast enough for near real-time damage monitoring. Prediction accuracy is generally superior to classical ML algorithms for image-based damage identification, often exceeding 95% in experimental studies under controlled laboratory conditions. The main advantages of CNN-based damage identification include automatic feature extraction from complex strain fields and high spatial resolution for crack localization. Nevertheless, CNN performance is strongly dependent on the availability of large labeled datasets, high-quality DIC measurements, and careful control of overfitting. In addition, limited generalization to new loading conditions and the high computational cost of training remain important challenges for practical engineering applications [187,188,189].

### 5.2. RNNs

RNNs and their advanced forms, such as long short-term memory (LSTM) and gated recurrent units, are well-suited for time-dependent problems and path-dependent material behavior. They have been applied to study anisotropic hardening and nonlinear plasticity in composites [190,191]. In damage detection, Sahoo and Jena [192] used RNNs to identify crack location and depth in graphene fiber-reinforced polymer (GFRP) beams from vibration data, achieving high accuracy. They used the first three natural frequencies obtained from FEM simulations of 20 cracked beams. The model achieved high accuracy with less than 2% error, but it was effective only for cracks large enough to noticeably reduce the beam’s stiffness. Zhi et al. [193] developed an RNN optimized with an extreme learning machine (ELM) to predict fatigue crack growth in aluminum alloys under variable loading, demonstrating the capability to model dynamic crack behavior. The model used the maximum and minimum cyclic stresses as inputs and the crack length as output. Feedback loops in the RNN helped it remember previous crack lengths and capture how the crack developed over time. From a physical viewpoint, RNN/LSTM architectures approximate nonlinear, history-dependent crack growth and damage accumulation laws rather than explicitly enforcing fracture mechanics-based models such as Paris-type relations (However, long-sequence prediction using RNNs is prone to error accumulation and drift, particularly when DIC or sensor-based time-series data are short, sparse, or noisy). The ELM method made the training fast and accurate by efficiently adjusting the model weights. Using experimental data from 2024-T351 aluminum alloy, the model accurately followed the real crack growth, proving its ability to predict time-dependent crack behavior effectively. Stocker et al. [194] integrated RNN-based constitutive models into FEM, improving softening and localization predictions for inelastic materials. Moreh et al. [195] proposed a hybrid RNN-CNN model for crack detection in large structures, achieving ~79% accuracy and demonstrating the potential of RNNs for SHM.

### 5.3. Autoencoder (AE)

AEs are feedforward neural networks that learn compressed data representations in an unsupervised manner, offering computational efficiency compared to nonlinear kernel principal component analysis. Recent studies have demonstrated their versatility in materials engineering and SHM. Jung et al. [196] applied a 3D convolutional AE with Bayesian optimization to design optimal microstructures, while Barile et al. [197] employed a deep AE for automated damage classification in CFRP composites. Moustakidis et al. [198] used an AE-based approach to compress acoustic emission signals, enabling accurate clustering of damage states in FRP composites. Li et al. [199] proposed a mechanics-informed AE for real-time SHM in civil infrastructure, and Shen et al. [200] integrated stacked denoising AE and convolutional denoising AE for multidimensional feature fusion in turbine blade crack detection. These works highlight the effectiveness of AEs in material design, automated damage detection, and SHM applications. However, excessive latent-space compression in AEs may suppress fine-scale strain localization and crack-tip singularity features, potentially leading to partial loss of mechanically critical damage information.

### 5.4. DBNs

DBNs are advanced neural architectures that use unsupervised learning across multiple stacked layers. Each layer typically consists of smaller neural networks that are often restricted Boltzmann machines. Also, while interlayer connections are present, nodes within a single layer are not interconnected. This hierarchical structure enables DBNs to effectively model complex data patterns and dependencies, making them suitable for feature learning, dimensionality reduction, and generative modeling. DBNs can be effectively applied to damage identification by learning hierarchical and abstract representations of sensor or image data. Through layer-wise unsupervised training (using Restricted Boltzmann Machines), DBNs automatically extract features that describe the normal behavior of a structure or material. Once trained, the network can differentiate between normal and defective states using supervised fine-tuning or by analyzing reconstruction errors when inputs deviate from learned patterns. This approach allows DBNs to handle complex, nonlinear, and high-dimensional data, such as vibration signals, ultrasonic waveforms, thermographic images, or DIC strain fields. By capturing the statistical distribution of “healthy” samples, DBNs can detect defects or anomalies like cracks, delaminations, or voids even under noisy conditions. Their generative ability also makes them suitable for anomaly detection, where defects are identified as samples that cannot be accurately reconstructed by the model. It should be noted that, despite their hierarchical representation capability, DBNs are currently less competitive than modern CNN and transformer-based architectures for high-resolution DIC strain-field analysis due to training instability and limited scalability [201,202,203].

### 5.5. GANs

GANs are a class of AI models designed to generate new data that closely resembles existing datasets, based on the interaction of two competing neural networks (a generator and a discriminator). Due to their ability to capture complex, high-dimensional distributions, GANs have gained increasing importance in materials science and SHM [204,205,206]. Jiang et al. [207] demonstrated their utility in inverse materials discovery, including composition design, processing optimization, crystal structure search, microstructure characterization, and damage identification. Similarly, Buehler [208] introduced a cycle-consistent GAN model capable of predicting atomistic stress fields from microstructures, thereby enabling physics-guided structural analysis.

GANs have also been applied to fracture and fatigue analysis. For example, in geological fracture networks, GANs have successfully reproduced realistic fracture geometries beyond the capacity of traditional models [209], while in hydraulic fracturing, GAN-based data augmentation addressed small-sample challenges, improving prediction accuracy of reservoir performance [210]. Physics-informed GANs have been developed to expand fatigue datasets, enhancing fatigue life prediction of 316L stainless steel by up to 91% [211]. In polymer composites, Helwing et al. [212] applied cycle-consistent GANs to generate synthetic fatigue damage states in computed tomography (CT) scans of fiber-reinforced polymers, enabling virtual damage augmentation and robust damage characterization.

More recent work has demonstrated GANs’ potential in NDT and damage identification. Tian et al. [213] enhanced GAN loss functions for crack detection in electromagnetic NDT, improving contrast and segmentation accuracy. Chen et al. [214] combined GAN-based crack image generation with CNNs and DeepLabv3+, significantly boosting detection performance when real datasets were limited. Similarly, GAN-based frame interpolation improved temporal resolution in laser welding, achieving near real-time (GPU-accelerated, laboratory-scale) defect tracking with >99% accuracy [215]. Modified GANs have also improved thermal image segmentation for crack detection in eddy current pulsed thermography [216].

Beyond fracture and defect monitoring, GANs are used for advanced material design. Yang et al. [217] integrated GANs with Bayesian optimization to improve microstructural design efficiency, while Hsu et al. [218] generated realistic 3D microstructures of fuel cell electrodes without requiring 3D imaging. Together, these studies illustrate how GANs enable not only defect detection and monitoring but also data augmentation, microstructure synthesis, and physics-informed material modeling, making them a powerful tool for next-generation materials research.

### 5.6. Deep Transfer Learning (DTL)

Deep neural networks typically require large datasets and significant computational resources for effective training. However, in many scientific domains, limited data availability presents a challenge. Deep Transfer Learning (DTL) addresses this issue by using pre-trained models on large, generic datasets to extract transferable features, enabling effective learning on smaller target datasets [201,219,220,221,222,223].

Recent studies of DTL in materials science demonstrate its importance. Dong et al. [224] combined DTL with a deep ANN, genetic algorithm, and Bayesian optimization for designing composite metal oxides, using the Magpie [225] descriptor. Li et al. [226], applied DTL achieving high accuracy with five labeled images, while Jia et al. [227] further advanced phase identification in superalloys.

Che et al. [228] developed a neural augmentation DTL model to improve fatigue damage evaluation in aircraft structures with limited data, enabling robust crack length prediction. Xiao et al. [229] used transfer learning with CNN and BP networks to predict the low-cycle fatigue life of corroded bimetallic steel bars. Liu et al. [230] integrated DTL with domain adaptation to transfer knowledge from simulations to experiments in CFRP composites, reducing experimental effort while maintaining high detection accuracy. Xu et al. [231] proposed a DTL-based model for CFRP health monitoring, mapping limited signal data to damage categories with high accuracy. Li et al. [232] applied DTL to construct allowable load spaces for notched laminates, improving generalization across design variations. Zhao et al. [233] used DTL for real-time localization of damage in composites from acoustic emission data, reaching 96.38% accuracy with reduced training time. Finally, Yazdani et al. [234] introduced a hybrid DTL model that combines EfficientNet and ResNet architectures to improve delamination detection in composite laminates using limited vibration-based SHM data. In their method, vibration signals from piezoelectric sensors were first converted into time-frequency scalogram images using the continuous wavelet transform. These images captured both temporal and spectral features of the vibrations. The hybrid model used EfficientNet to extract multi-scale spatial features efficiently, while ResNet captured deeper hierarchical patterns through residual learning. The combined model was fine-tuned on experimental data from composites with three health conditions (healthy, D1, and D2 delamination). Results showed that the hybrid model achieved higher accuracy and robustness than using either network alone, effectively identifying delamination even with a small dataset.

DL models offer computationally powerful but conditionally reliable tools for defect and anomaly identification across a variety of engineering and industrial applications. CNNs and DBNs are mainly used for supervised classification of defect features, while RNNs are effective for analyzing temporal data and identifying developing faults. AEs and GANs function primarily in an unsupervised manner, learning the normal system response and detecting anomalies through deviations in reconstruction or discriminator outputs. These models enable automatic feature learning and enhanced defect localization, while their operational reliability remains strongly conditioned by data quality, domain shift, physical consistency, and uncertainty-aware validation. Table 3 presents a summary of DL methods for damage assessment in engineering materials.

Overall, the selection of an appropriate ML/DL algorithm for damage identification depends on the size and nature of the dataset, real-time requirements, and available computational resources. SVM and k-NN are suitable for small datasets with engineered features, offering low training cost but limited scalability. Decision Tree and ensemble methods provide good interpretability and robustness for medium-sized datasets. In contrast, CNNs and deep architectures achieve the highest accuracy for DIC-based image and strain field analysis but require extensive optimization, large labeled datasets, and high computational power. Therefore, real-world monitoring requires a careful balance between accuracy, speed, and practical use [187,241].

Unlike conventional computer vision problems, DIC-based learning relies on physics-driven displacement and strain fields that exhibit strong spatial continuity, scale dependency, and sensitivity to experimental noise. These characteristics fundamentally distinguish DIC-ML integration from generic image-based damage detection and necessitate dedicated data preprocessing, strain-aware network design, and physics-consistent validation [20,157].

Despite the high predictive accuracy reported for many DL architectures, uncertainty quantification is rarely addressed in current DIC-driven DL frameworks. Bayesian neural networks, Monte Carlo dropout, and ensemble-based uncertainty estimation provide practical tools to quantify pixel-wise segmentation uncertainty in crack detection and confidence bounds in damage-state classification from DIC strain maps, which is essential for safety-critical SHM applications [16,242,243,244]. In future DIC-ML frameworks, uncertainty-aware damage indices that combine pixel-wise segmentation probability with strain-field confidence bounds are expected to play a key role in defining reliable decision thresholds for safety-critical structures.

## 6. Use of DIC and ML for Damage Assessment

**Figure 4:**
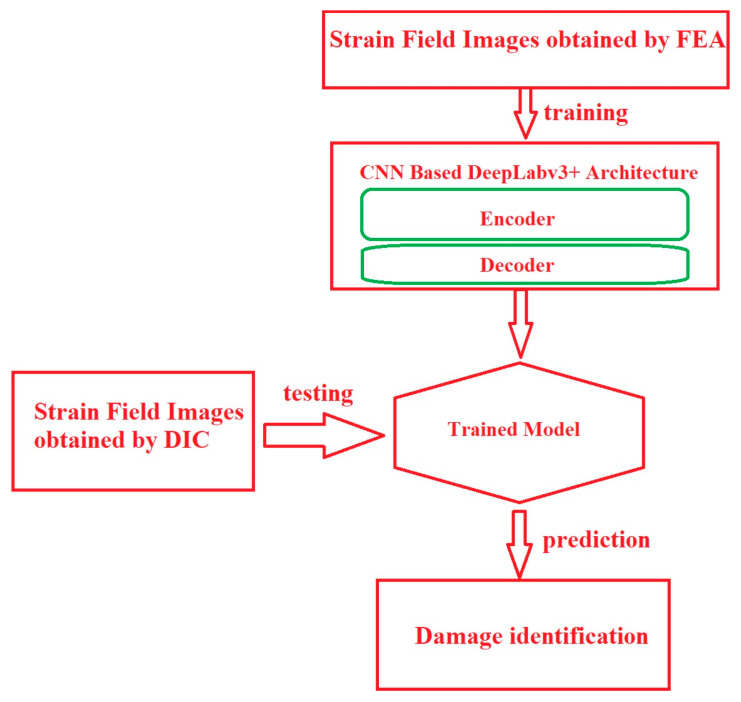
Overview of the proposed ML-assisted DIC workflow for damage assessment used in Ref. [245].

Wang et al. [245] developed an ML-assisted DIC framework for automated damage detection in CFRP laminates. In their methodology, DIC was first employed to obtain full-field surface strain distributions of specimens subjected to quasi-static tensile loading. Since the performance of supervised semantic segmentation strongly depends on the availability of large labeled datasets, finite element analysis (FEA) was utilized to generate a sufficient number of strain field images with known damage states for the training and validation phases (as shown in Figure 4). The use of FEA allowed efficient generation of diverse damage scenarios with accurate ground-truth labels while significantly reducing experimental cost and time, although this may introduce a simulation-to-experiment domain shift when transferring trained models to real DIC measurements. The strain field images obtained from FEA were normalized, resized, and labeled at the pixel level into three semantic classes, namely background (healthy region), open-hole damage, and damage initiation. These labeled images were then augmented through translation and reflection to further increase dataset diversity and avoid overfitting. The augmented strain images were subsequently used to train a DeepLabv3+ semantic segmentation model. The DeepLabv3+ architecture was composed of an encoder and a decoder. The encoder, based on a pre-trained ResNet-50 backbone combined with an atrous spatial pyramid pooling (ASPP) module, was used to extract high-level multiscale features from the strain field images. The ASPP enabled the network to capture damage-related features at different spatial scales. The decoder was then used to upsample the encoded feature maps and fuse them with low-level spatial features in order to accurately recover the boundaries of damage regions at the pixel level. The network was trained using the stochastic gradient descent optimization algorithm by minimizing the prediction error between the segmented output and the ground truth labels. After training, the experimental DIC strain field images, which had not been used in the training process, were fed into the trained network during the testing phase. The trained model then produced pixel-level segmentation results that automatically identified and localized damaged regions with high accuracy under controlled experimental conditions and dataset-dependent robustness in the CFRP specimens.

However, the uncertainty associated with DIC measurements (including speckle noise, subset size dependency, and strain differentiation error) is implicitly transferred into the ML prediction stage, yet most existing studies do not explicitly quantify or propagate this uncertainty into the final damage decision.

ML for damage assessment using DIC focuses on recognizing, classifying, and predicting the presence and evolution of damage within materials based on the spatial distribution of strain and displacement fields. DIC provides full-field measurements of deformation, which contain subtle indicators of material degradation long before visible cracks appear. By applying ML, these complex patterns can be automatically analyzed to identify damaged regions with accuracy that strongly depends on data quality, noise level, and training domain. The general idea is to train an algorithm to distinguish between undamaged and damaged states from DIC data. During experiments, DIC captures surface deformation maps of specimens under different load levels. These strain fields are transformed into structured data that serve as inputs to a learning model. Each map or subregion is labeled according to whether damage exists (based on ground-truth observations from microscopy, X-ray, or human inspection). The goal of the ML model is to learn the underlying patterns that separate intact areas from damaged ones. Preprocessing the DIC data is a vital step. Noise filtering and normalization ensure that variations due to lighting, speckle pattern density, or camera noise do not mislead the model. Often, the DIC strain field is divided into smaller subregions (patches) so that the model can analyze local deformation behavior. In each subregion, features are extracted to represent the mechanical condition [20,165,245,246].

Traditional feature-based methods compute descriptive statistics such as the mean, standard deviation, and maximum strain, as well as spatial gradients, strain energy density, or localized strain concentration factors. These engineered features transform complex strain maps into lower-dimensional numerical representations suitable for classical ML models. For damage identification using these feature-based approaches, algorithms such as Support Vector Machines (SVM), Random Forests, Decision Trees, and Gradient Boosting (XGBoost) are commonly employed. Compared with feature-based ML models such as SVM, Random Forest, and XGBoost, CNN-based architectures operate directly on full-field DIC strain maps without manual feature engineering, enabling pixel-level damage localization. However, classical ML models remain computationally more efficient and better suited for small DIC datasets with engineered strain descriptors. The SVM, for instance, can effectively find nonlinear boundaries between damaged and undamaged states by using kernel functions. Random Forests and XGBoost can handle large feature sets, manage noise, and provide feature importance analysis, revealing which strain components are most sensitive to damage initiation. These models are trained using labeled DIC datasets, where the input features are strain-related descriptors and the output label is the damage class (for example, healthy, minor damage, or severe damage). DL can become especially effective for DIC-based damage identification. Instead of manually designing features, CNNs automatically learn discriminative spatial patterns directly from DIC strain or displacement maps. CNNs apply convolutional filters to extract hierarchical features that represent texture, orientation, and concentration patterns linked to cracks or local stiffness loss. When the task requires locating damage precisely, fully convolutional models such as U-Net or SegNet are applied to produce pixel-level segmentation maps, indicating the exact regions of damage. In DIC-based segmentation, severe class imbalance between damaged and undamaged pixels represents a major learning challenge, often requiring weighted loss functions, focal loss, or data resampling strategies to avoid biased predictions [20,165,245,246,247,248].

These networks can detect even microscopic cracks by recognizing the strain concentration signatures characteristic of material failure. Training the model involves dividing the DIC dataset into training, validation, and test subsets. The model parameters are optimized to minimize a loss function (cross-entropy for classification or Dice/IoU loss for segmentation) using algorithms such as Adam or stochastic gradient descent. Proper regularization, dropout, and data augmentation (rotations, scaling, and brightness changes) are used to improve generalization. Evaluation metrics such as accuracy, precision, recall, the F1-score, and the area under the ROC curve are calculated to assess how well the trained model identifies damage [20,247].

Interpretability remains important in damage identification. Feature-based models allow examination of which strain features contribute most to predictions, helping engineers link the model’s output to physical mechanisms of damage. DL models can be interpreted using visualization tools such as Grad-CAM or saliency maps, which highlight the regions of the strain field that influenced the model’s decision. This ensures that the model’s reasoning aligns with mechanical understanding (for example, focusing on areas with strain concentration or shear localization rather than irrelevant regions). ML enables near real-time damage identification during DIC experiments depending on camera resolution, strain computation speed, and network inference cost. Instead of manually inspecting strain maps, the trained model can instantly flag regions of potential damage as the test progresses. It should be noted that DIC-ML frameworks exhibit higher reliability for crack-driven and strain-localization-dominated damage, whereas diffuse damage mechanisms such as micro-void coalescence or subsurface delamination remain more challenging for surface-based DIC sensing [16,242,247]. This capability enhances structural health monitoring, early failure prediction, and quality control in materials testing. Ultimately, using DIC with ML transforms raw deformation measurements into intelligent diagnostic information, making the process of damage identification faster, more objective, and more predictive.

DIC provides dense full-field displacement and strain measurements that fundamentally differ from conventional grayscale images or low-dimensional sensor signals typically used in structural health monitoring. These data exhibit strong spatial correlation, scale dependency, and physics-driven continuity, which directly influence the learning behavior of ML and DL models. Unlike natural images, DIC strain maps often contain noise induced by speckle quality, lighting variation, and subset correlation errors, requiring dedicated preprocessing such as spatial filtering, multiscale decomposition, or strain-window normalization. Failure modes of DIC-ML systems include loss of strain continuity due to poor speckle quality, over-smoothing of strain gradients during filtering, misclassification under changing lighting conditions, and performance degradation under unseen loading paths [20,157,249]. These limitations highlight the need for physics-informed constraints and uncertainty-aware learning strategies.

From a learning perspective, DIC data enable pixel-level damage segmentation, strain-gradient-based feature extraction, and crack-tip localization with subpixel resolution. CNN-based architectures are particularly effective for capturing localized strain concentrations and discontinuities associated with crack initiation and delamination. However, the physics-driven nature of DIC fields also poses challenges, including strong class imbalance between damaged and undamaged regions, sensitivity to experimental noise, and limited availability of large labeled strain datasets. These DIC-specific constraints distinguish ML-based damage identification from conventional vision-based object detection and necessitate tailored network architectures and training strategies [157,187,250].

Furthermore, the temporal continuity of DIC measurements enables time-series learning for damage evolution and fatigue crack growth analysis. Recurrent models and temporal CNNs can exploit sequential strain-field information to predict damage progression, offering a pathway toward physics-informed and prognostics-oriented DIC-ML integration.

**Figure 5:**
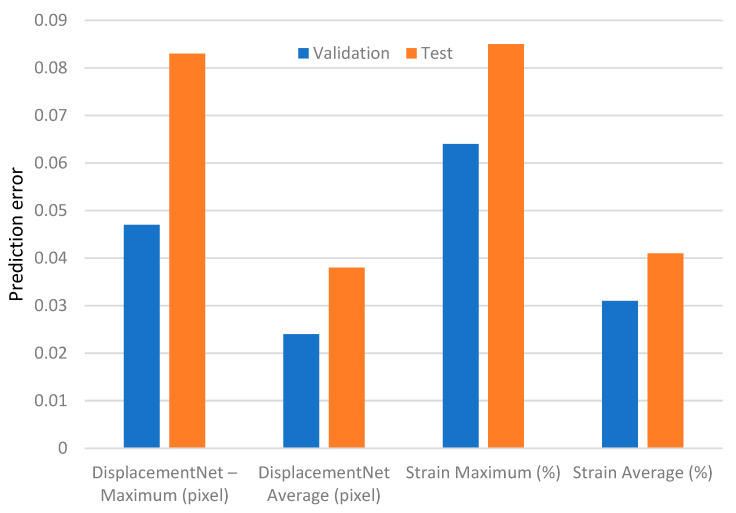
Evaluation results of Deep DIC on both the validation and test datasets [157].

To quantitatively evaluate the predictive capability and generalization performance of DL-based DIC, Yang et al. [157] assessed their proposed Deep DIC framework using synthetic speckle images with known ground-truth displacement and strain fields. Synthetic datasets are commonly adopted in DL-based DIC studies because they provide fully controlled deformation fields, allowing precise error quantification and systematic performance assessment under different deformation levels. After training, the DisplacementNet and StrainNet models were evaluated on an independent test set of 150 synthetic image pairs that had not been used during either the training or validation stages, providing an unbiased measure of the model’s predictive ability on unseen data. In addition to the final evaluation on unseen samples, the study also examined the model’s accuracy on the validation data used during training to better characterize its behavior across familiar and unfamiliar deformation patterns. For the validation set, the displacement errors were low—0.047 (maximum) and 0.024 pixels (average). The strain errors were similarly small, with maximum and average values of 0.064% and 0.031%. When applied to the unseen test data, the error levels increased moderately, as expected for entirely new deformation fields. The maximum and average displacement errors rose to 0.083 and 0.038 pixels, representing increases of about 76% and 58%. The strain errors increased to 0.085% and 0.041%, corresponding to increases of approximately 33% and 32% (absolute percent strain). These results, visualized in Figure 5 through a bar-chart comparison, show that although the test errors are slightly higher, all values remain low—within sub-pixel accuracy for displacement and below 0.1% for strain. The close match between validation and test performance indicates strong generalization and stable predictive accuracy of the Deep DIC framework.

To further examine the practical reliability of the method, Yang et al. [157] selected two representative test cases corresponding to relatively small and large deformation levels and compared the predicted displacement and strain fields with both the ground truth and the results obtained from the commercial DIC software VIC-2D (v6, 2021, Correlated Solutions, Inc., USA) (Test 1 represented a small but complex deformation case with localized strain gradients and noticeable shear, while Test 2 corresponded to a large and smooth tensile deformation with negligible shear.). For a fair comparison, both examples were processed in VIC-2D using a subset size of 7 pixels and a step size of 2 pixels. Since the output resolution of VIC-2D is smaller than that of the original input images, the VIC-2D results were interpolated to a resolution of 128 × 128 using MATLAB interpolation functions *interp2* to ensure consistency with the Deep DIC and ground-truth data. The average prediction errors of the two displacement components and three strain components were then computed for both Deep DIC and VIC-2D. The quantitative comparison of these two approaches is presented in Table 4. The results show that the Deep DIC framework achieves prediction accuracy comparable to, and in some components even better than, the commercial VIC-2D software, particularly under large-deformation conditions. This superiority is also quantified in Table 4, which reports the relative improvement (%) of Deep DIC over VIC-2D. Specifically, Deep DIC reduces the displacement error by 76.9% and 86.1% for the two displacement components in Test 1, and achieves 68.8%, 93.0%, and 48.7% improvement for the three strain components. In the large-deformation Test 2, the improvements are even more significant, with 85.5% and 81.9% reduction in the displacement-component errors and 68.8%, 82.1%, and 58.4% enhancement in the strain-component accuracy. These numerical results further confirm the advantage of Deep DIC in both small-complex and large-simple deformation fields. It should be noted that Test 1 and Test 2 are not physical mechanical tests, but rather two synthetic deformation cases extracted from the test dataset and used solely to evaluate the accuracy of Deep DIC under small-complex and large-simple deformation patterns.

Although this section is organized based on material categories, it should be emphasized that this classification is not driven by differences in DIC data acquisition, since surface morphology images and strain fields of both material systems are obtained using identical optical principles. Rather, this categorization is mainly motivated by the distinct failure mechanisms associated with different materials. Composite materials are commonly characterized by matrix cracking, fiber breakage, and interlaminar delamination, whereas metallic materials are dominated by crack initiation and propagation, fatigue damage, and plastic strain localization. These different failure mechanisms give rise to characteristic strain-field patterns in DIC measurements, which in turn affect the suitability and performance of specific ML and DL models [245,246].

### 6.1. The Use of DIC and ML for Damage Assessment in Composites

For composite materials, DL models are mainly trained to recognize DIC-based strain features associated with delamination, matrix cracking, fiber fracture, and barely visible impact damage, which typically produce discontinuous and highly heterogeneous strain patterns. These materials, such as CFRPs and woven laminates, are widely used in aerospace and structural systems for their high specific strength and designed anisotropy. However, their complex damage mechanisms (fiber fracture, matrix cracking, delamination, and barely visible impact damage) make traditional non-destructive methods inadequate [71,251]. The use of DIC with ML emerges as an effective strategy for automated, high-resolution detection and interpretation of damage in these materials.

Several studies have highlighted the power of combining DIC-generated strain fields with DL. Wang et al. [245] applied DeepLabv3+ for semantic segmentation of barely visible impact damage in CFRPs. Chi et al. [186] introduced StrainNet-LD, a CNN-based method that achieved subpixel accuracy and real-time large displacement analysis. Cidade et al. [252] integrated ML with DIC to compute mode I fracture toughness in composites. Ma et al. [253] and Niu et al. [254] improved DIC reliability under poor speckle conditions using super-resolution CNNs and denoising models. Zhou et al. [255] demonstrated that transformer-based models outperform CNNs in capturing anisotropic strain fields, while Akgun et al. [256] combined stereo-DIC and CNNs to localize shear bands and delamination in CFRP cylinders under compression-torsion. In Ref. [246], the trained YOLOv5x model was deployed for online crack segmentation during tensile testing. As the DIC system continuously generated Sigma maps during loading, these images were fed into the neural network in real time. The model automatically segmented transverse and longitudinal cracks, assigning bounding boxes and class labels with high accuracy throughout the loading process. At the initial loading stage, when cracks were few and clearly separated, the crack recognition and segmentation accuracy reached nearly 100%. As loading progressed, the number and density of cracks increased and complex crack intersections developed, which slightly reduced the prediction accuracy, however, the segmentation performance consistently remained above 95%. After full training, the YOLOv5x model achieved a mean average precision of 98.7%, with a transverse crack detection accuracy of about 99% and longitudinal crack accuracy of about 95%. These results confirmed that the DIC-YOLOv5x system functioned effectively as a real-time, non-contact structural health monitoring tool. Using the ML-based crack segmentation results, the researchers performed quantitative damage analysis by statistically tracking the crack number throughout loading. The crack evolution curves were directly correlated with the mechanical response of the composites. It was observed that the tangent modulus progressively decreased with increasing surface crack density, confirming that surface crack accumulation governed stiffness degradation. Furthermore, at final failure, specimens subjected to different aging durations exhibited a similar total number of surface cracks, indicating that the damage threshold was governed mainly by the periodic woven architecture rather than aging time alone. Thus, DIC provides accurate full-field strain and damage data, while ML enables automated crack identification, classification, and statistical quantification. Their integration established a direct quantitative relationship between mechanical degradation and real-time surface damage evolution, enabling true online failure monitoring of 3D woven composites.

Table 5 summarizes recent studies on composites using ML with DIC for damage assessment in composite materials.

Mechanics-informed neural networks trained on DIC strain fields [257] reproduced anisotropic elastic responses with errors on the order of 10^−4^, showing how ML can complement DIC for constitutive modeling. Beyond polymer composites, recent work has extended DIC-ML combinations to hybrid and repaired systems. Gao and Deng [258] studied mixed-mode fatigue in steel plates repaired with CFRP overlays, using DIC for full-field strain tracking and ML for interpreting crack growth patterns. Their results showed that CFRP reinforcement significantly delayed crack propagation and enhanced fatigue life. Tian et al. [259] investigated steel-UHPC composite beams under flexural loading, combining DIC with ML-enhanced acoustic emission analysis to detect interface cracking and track progressive damage in real time. Song et al. [260] focused on pre-corroded Al-Li alloys, for which ML models trained on DIC-derived features improved fatigue life predictions compared to traditional empirical methods, while DIC provided insight into crack initiation and localized deformation.

Together, these studies demonstrate that integrating DIC with ML not only enhances the precision and robustness of damage detection in CFRPs and woven composites but also extends to repaired metallic-composite systems and corroded alloys. This approach offers powerful, data-driven tools for monitoring, predicting, and interpreting complex anomaly processes across advanced composite structures.

These studies clearly demonstrate the value of combining DIC and ML for accurate, automated, and early-stage damage identification in composite materials under diverse mechanical loading conditions.

### 6.2. Using DIC and ML for Identification of Damage in Metallic Materials

For metallic materials, ML and DL techniques are primarily applied to detect crack initiation, crack propagation, fatigue damage, and plastic strain localization, which generate relatively continuous and concentrated strain features in DIC fields. Metallic materials such as aluminum alloys, steels, and stainless steels are essential to structural and transportation systems but are highly susceptible to fatigue, plastic deformation, and residual stress. The combination of DIC with ML has emerged as a powerful strategy for automated damage identification, crack monitoring, and life prediction. Early work demonstrated that DL models such as ParallelNets [261], ResNet [262], and U-Net [247,261] could achieve sub-millimeter accuracy in crack tip localization and path segmentation. CNNs have also been applied to extract stress–strain curves in stainless steels [263], detect bolt loosening in steel joints [264], and identify additive manufacturing damages in real-time with >95% accuracy [265]. Expanding these applications, CNNs have been used for structural health state classification with 99% accuracy [266], von Mises stress reconstruction under biaxial loading [157], mixed-mode SIF extraction using brittle metal analogs [267], and uncertainty-aware strain prediction [268]. Other advances include unsupervised CNNs for direct displacement mapping [269], hybrid FEM-DIC models for internal stress prediction in adhesives [270], and CNN-LSTM control systems for improving thermomechanical forming processes [271].

In addition, generative and adaptive ML approaches have further expanded the use of DIC. A physics-guided GAN was introduced to synthesize displacement fields constrained by von Mises strain, improving data generation for crack models [272]. Self-supervised learning models have enabled crack detection from 3D-DIC velocity data under heat distortion and large deformations [273]. Gaussian Process Regression-based methods, such as the Inflection Point Method (IPM), have been combined with line-based DIC to provide smooth, automated crack length tracking in steels and aluminum alloys [274]. Regression models trained on normalized deformation profiles have been employed to predict surface-breaking crack depth robustly across different geometries and load cases [275]. DL models such as CrackNet have been used to localize crack tips in large-scale steel plates under fatigue loading with high accuracy and minimal manual input [276]. Finally, studies on thermo-mechanical fatigue in steels showed that combining DIC with ML improves the characterization of short crack growth sensitivity to varying temperature-load phase angles [277].

Overall, the combination of DIC with ML (from supervised and unsupervised CNNs to generative and regression-based models) has significantly advanced the identification of damage in metallic materials. These approaches not only improve accuracy and automation in crack monitoring but also extend to stress reconstruction, structural health classification, process optimization, and lifetime prediction, underscoring their growing importance in next-generation SHM systems. Strohmann et al. [247] proposed an automated method for detecting fatigue crack paths and crack tip positions by combining DIC with a CNN. Fatigue crack propagation experiments were conducted on AA2024-T3 aluminum alloy specimens, and full-field displacement data obtained from DIC were used as direct inputs to a U-Net-type CNN for segmenting the crack path and crack tip. The network was trained using manually labeled experimental data and additional displacement fields generated by finite element simulations. The trained model accurately reproduced crack growth (a-N) curves and reliably detected crack evolution for specimens with different geometries. The mean crack-tip position error remained below 0.5 mm for the main specimen and about 3-4 mm for a different specimen. They concluded that ML enabled fully automated, objective crack tracking directly from displacement fields, significantly reducing manual post-processing and improving robustness against optical noise.

These studies collectively highlight the growing potential of DIC-ML systems in monitoring, diagnosing, and predicting failure in metallic structures under a wide range of loading and environmental conditions. In the following, a summary of some studies reviewed in this section is presented in Table 6:

### 6.3. Other Engineering Materials

Beyond composites, metals, and graded structures, the dominant damage characteristics (such as tensile crack initiation, mixed-mode crack propagation, porosity formation, and distributed microcracking) govern the feature patterns observed in DIC strain fields and therefore determine the suitability of specific ML architectures, particularly DL models, rather than the material class itself. In these materials, the combination of DIC with ML has been extended to a wide range of engineering materials, including sandstone, asphalt, concrete, cement-based systems, and even biological tissues, where heterogeneous deformation and complex crack evolution pose major challenges for traditional inspection methods. In additive manufacturing, Gaussian Process Regression has been used to optimize Laser Powder Bed Fusion parameters for metal matrix composites, improving strength and reducing damages with limited experimental datasets [280]. DL approaches, such as U-Net, YOLO, and Mask R-CNN, have been trained on thermal, X-ray, or infrared data to detect porosity, spatter, and melt pool irregularities in real-time [281], while YOLOv5x achieved >92% accuracy in detecting powder bed anomalies [282].

**Figure 6:**
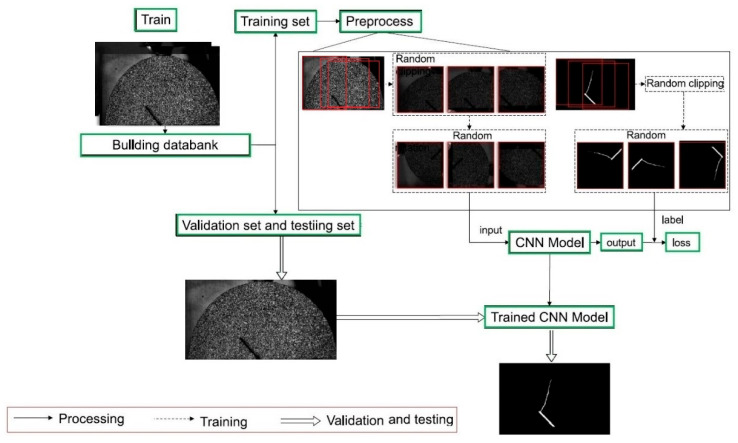
ML based damage segmentation workflow [267].

Zhang et al. [267] studied a novel method for the automatic identification of cracks and crack tips in sandstone by combining a DL model (U-Net) with the DIC technique. According to the authors, the overall workflow of the ML procedure used for crack segmentation and detection can be seen in Figure 6. The process begins with building a databank of images collected from three-point bending experiments. These images were captured continuously by a CCD camera during the loading of semi-circular sandstone specimens, showing the gradual propagation of cracks. The databank was then divided into two parts: a training set and a validation and testing set. Before training, the images in the training set were preprocessed to enhance their quality and diversity. The preprocessing included random clipping, where large original images were cropped into smaller regions to increase the number of training samples and allow the model to learn local crack features. Random rotation was also applied to the images so that the model could become robust to cracks of different orientations. In addition, histogram equalization was used to improve contrast and make cracks more distinguishable from the background. Each input image was paired with a manually labeled ground-truth image, in which the cracks were marked in white and the background in black. These paired datasets formed the input-label structure required for supervised training. The prepared dataset was then provided as input to a convolutional neural network, specifically a U-Net architecture. The CNN received the preprocessed image as input and produced a predicted segmentation map as output. The predicted output was compared with the labeled ground truth, and the difference between the two generated a loss value. This loss function guided the backpropagation process to iteratively adjust the model’s weights, gradually improving the network’s ability to identify cracks accurately. Through repeated training cycles, the model learned to distinguish crack pixels from background noise and achieved high segmentation accuracy. After training, the model was validated and tested using the separate validation set. During this stage, unseen images were input into the trained CNN model to evaluate its performance. The output was a binary segmentation image showing the detected crack as a white region against a black background. The comparison between the predicted and actual crack shapes verified the model’s effectiveness.

**Figure 7:**
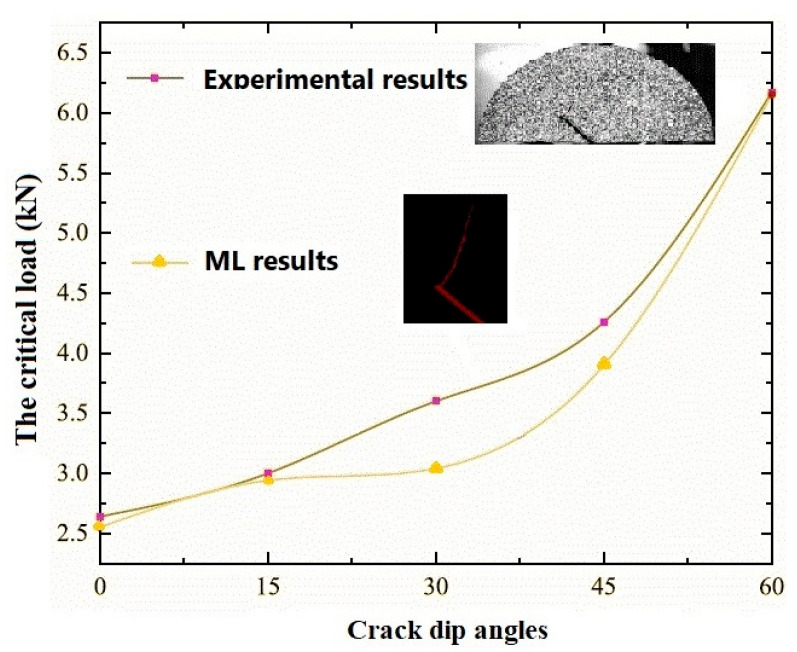
Comparison of ML and experimental results for critical loads versus crack dip angles [267].

In three-point bending experiments, semi-circular specimens with different crack inclination angles (0°, 15°, 30°, 45°, and 60°) were tested. The surfaces of the specimens were coated with a random speckle pattern, and during loading, a CCD camera continuously captured sequential images of the specimen surface. The DIC method compared the deformation of these speckles in images taken before and after loading, thereby extracting the surface displacement and strain fields. To detect cracks automatically, a U-Net convolutional neural network was trained to recognize cracks in the DIC images. The training dataset consisted of preprocessed images enhanced by histogram equalization and manually labeled cracks. After training, the U-Net achieved an accuracy of approximately 99%, successfully distinguishing crack pixels from the background and automatically identifying the crack tip position in each frame. The coordinates of these crack tips were then used to analyze crack growth behavior over time and to calculate the stress intensity factors (SIFs) for Modes I and II. The U-Net output data were used together with the DIC results to perform stress analysis. The automatically identified crack tip coordinates were substituted into the stress field equations based on the Williams expansion to obtain the corresponding SIF values for each crack angle and loading stage. The results showed that for smaller crack angles (0° and 15°), the crack propagated primarily under tensile Mode I. At medium angles (30° and 45°), the propagation exhibited mixed-mode (I-II) behavior, and at 60°, the crack growth was dominated by shear Mode II. Figure 7 presents the variations in the critical load for different crack inclination angles. In this figure, the curves derived from the U-Net model are compared with experimental data. The strong overlap between the two curves demonstrates that the model’s predictions closely match the experimental results, indirectly confirming the high accuracy of the U-Net in identifying crack tips and determining the critical load.

Similarly, ML models trained on temporal DIC strain sequences detected early asphalt crack initiation up to 25% faster than visual inspection [283]. Comparative studies [284] confirmed the advantage of DL-based crack segmentation, with pretrained TransNet models significantly improving precision compared to thresholding. In concrete structures, Gharehbaghi et al. [189] achieved 92.6% detection accuracy using R-CNNs on 2D-DIC strain fields of reinforced beams under heavy loads, while Zhu et al. [285] introduced a panoramic multi-camera DIC system combined with ML-based classification to monitor large-scale concrete beams, achieving accurate crack localization across wide fields of view.

Extending beyond conventional engineering materials, Tragoudas et al. [286] applied DIC and a DL model (attention residual U-Net with Monte Carlo dropout) to vascular tissue fracture analysis. Using porcine aorta specimens under compact tension loading, their method replicated high-resolution DIC displacement fields and enabled automated crack evolution tracking, even under noisy and limited data conditions. Notably, the approach supported inverse identification of fracture parameters for FEM simulations, highlighting the potential of DIC-ML combinations for biomedical fracture mechanics.

These studies show that coupling DIC with ML enables accurate, automated, and scalable crack identification across diverse materials (from AM alloys and asphalt pavements to reinforced concrete and vascular tissues) and highlight its growing importance in SHM and other applications.

**Figure 8:**
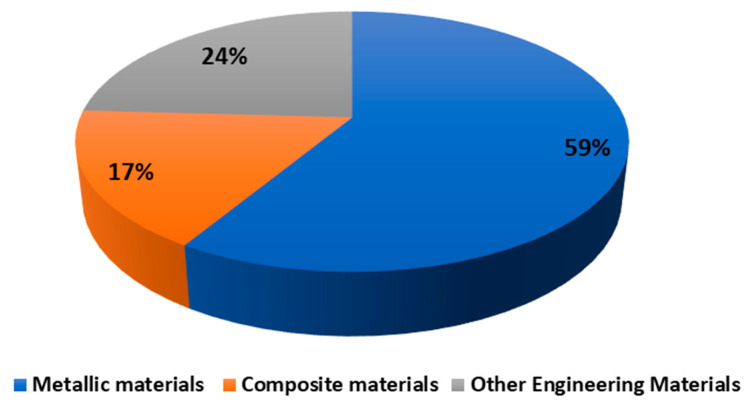
Proportion of materials used in this review paper employing ML with DIC for damage assessment.

Considering the previous studies reviewed in this paper, the proportion of papers that investigated damage using ML and DIC in metallic, composite, and other materials can be compared and depicted in Figure 8. From this figure, it can be noticed that metallic materials represent the largest portion (≈58.6%) of studies combining ML and DIC because metals are the most extensively used materials in engineering structures, and their fatigue, crack initiation, and fracture behaviors have been studied for decades. The large amount of experimental data available and the relatively straightforward surface preparation make it easier to apply DIC and train ML models accurately. Metals also play an important role in SHM, which motivates researchers to adopt ML-DIC systems for automated crack identification, stress reconstruction, and fatigue life prediction. In addition, other engineering materials, such as concrete, rock, asphalt, and biological tissues, account for about 24.1%. Their share is growing because DIC is particularly useful for brittle or heterogeneous materials, where conventional sensors fail to capture full-field deformation. However, these materials often exhibit complex and irregular fracture patterns, and collecting consistent data is more difficult, which limits the number of available studies. Finally, composite materials make up the smallest proportion (≈17.2%) due to the complexity of their microstructures and anisotropic behavior. Applying DIC on composites requires high-quality speckle patterns and careful experimental setups, and their heterogeneous strain fields generate noisy data that are challenging for ML algorithms to interpret. Using ML-DIC for composites is a rapidly advancing field, especially with the rise in DL and semantic segmentation models for identifying delamination and fiber breakage.

ML techniques have recently been widely integrated with full-field experimental measurement methods to enable automated and quantitative damage analysis in materials and structures. Among these techniques, DIC has emerged as a key experimental tool for providing physically meaningful displacement and strain data for data-driven damage detection. The reviewed studies clearly indicate that the success, accuracy, and physical reliability of ML-based damage analysis are fundamentally dependent on the availability of high-quality DIC measurements. In ref. [246] without the use of DIC, real-time monitoring of crack initiation and progressive damage evolution would not have been possible, and the damage-sensitive Sigma parameter used as the primary input to the YOLO model would not have been available. Consequently, early microcrack detection, the quantitative correlation between stiffness degradation and crack accumulation, and the high prediction accuracy of the DL model would have been severely compromised. This dependence on DIC is consistent with ref. [245], where the deep-learning-based semantic segmentation framework relied entirely on DIC-derived full-field strain maps. Without these data, damage initiation based on strain singularities and the detection of barely visible damage would not have been feasible, and the analysis would have been limited to post-damage visual inspection rather than true, online structural health monitoring.

A similar level of dependence on DIC was observed in ref. [157], in which both displacement and strain fields were learned directly from speckle image pairs acquired during deformation. All key capabilities of the framework, including end-to-end full-field prediction, direct strain estimation without numerical differentiation, and adaptive region-of-interest tracking, were fundamentally rooted in DIC imaging. Without DIC, the physical validity of the deformation predictions and their verification against commercial systems would not have been possible. Beyond damage localization, DIC also played a decisive role in fracture mechanics-based ML applications. In ref. [252], full-field displacement and strain data obtained by DIC were essential for the experimental evaluation of the dynamic J-Integral and for the direct determination of dynamic fracture toughness under high strain-rate loading. Without DIC, both the validation of the inertia-sensitive J-Integral formulation and the ML-based sensitivity analysis of DIC parameters would have been infeasible, reducing the study to indirect or simulation-based estimations.

In ref. [247], the CNN for automatic crack path and crack tip detection relied exclusively on DIC-measured displacement fields rather than surface appearance. This allowed automated construction of crack growth (a-N) curves and validation against fracture mechanics parameters. Without DIC, the approach would have reverted to noise-sensitive, purely visual crack detection, and quantitative fatigue crack monitoring would not have been achievable. Finally, in ref. [284], DIC-derived maximum principal strain maps formed the basis of both threshold-based and deep-learning-based crack segmentation. Without DIC, strain-based crack detection, the comparison between conventional thresholding and DL, and the physics-based evaluation of crack maps for structural damage assessment would not have been possible, restricting the analysis to purely visual crack identification. Overall, these studies consistently demonstrate that DIC is not merely an auxiliary imaging technique but a core experimental enabler that supplies physically grounded inputs for ML. The integration of ML with DIC transforms damage assessment from a subjective, visual procedure into a quantitative, full-field, and physics-based analysis framework. Without DIC, most ML approaches reviewed here would lose their physical relevance and be reduced to qualitative inspection tools with limited reliability.

On the other hand, not all ML-based damage analysis studies have relied on DIC measurements because the research objectives did not require full-field experimental displacement or strain measurements. For instance, in ref. [287], crack detection was performed using only statistical texture features extracted from surface images, and the goal was to achieve fast and robust visual crack onset classification without dealing with correlation-related uncertainties of DIC. Therefore, the methodology was purposely designed as a purely image-based framework. Likewise, in ref. [97], the entire analysis was conducted in a purely numerical context using phase-field fracture simulations, and ML was applied as a surrogate model to accelerate computationally expensive crack propagation predictions. Since all data were generated numerically and no experimental measurements were involved, the use of DIC was not required for the intended analysis. However, the absence of DIC in both studies also limited the physical interpretation of their results. If DIC had been employed, ref. [287] could have enabled early crack detection based on strain localization rather than surface appearance alone and allowed quantitative crack growth tracking. In ref. [97], the incorporation of DIC would have provided experimental validation of the ML-based crack propagation predictions under real loading conditions. Thus, although DIC was not necessary for the original objectives of these works, its integration could have significantly enhanced the physical relevance and experimental validation of their ML-based damage analyses. Also, in [71], DIC was not employed because the primary objective was limited to the detection and geometric characterization of visible surface cracks using conventional optical images and DL. The methodology was intentionally designed as a purely image-based framework, in which crack features were extracted directly from RGB or grayscale images without the need for full-field displacement or strain measurements. Since no continuous loading history, displacement tracking, or strain-based damage indicators were required for the proposed analysis, the use of DIC was not necessary for achieving the intended objectives of the study.

It should be noted that the apparent separation between composite, metallic, and other materials in this section is essentially driven by their dominant failure characteristics rather than by differences in DIC imaging itself. From a data-driven perspective, ML architectures, particularly DL models, are therefore selected according to the specific damage features to be identified (for example delamination versus crack growth), independent of the material category.

## 7. Observation, Challenges, and Future Research Directions

AI-based approaches have demonstrated promising results, offering valuable insights in modeling the mechanical behavior of composites. Traditional ML methods, such as SVM and ANNs, have shown strong predictive accuracy, often aligning well with experimental data and simulations. These models can effectively learn from limited data and capture complex, nonlinear relationships without prior assumptions. Table 7 summarizes the strengths and limitations of common traditional ML techniques used in this domain. Although traditional ML methods have achieved promising results, they still face some drawbacks when applied to composite materials. They depend heavily on manual feature design, have limited ability to capture complex material behavior, and their multi-step process often lowers overall accuracy and efficiency.

DL methods, a specialized branch of ML, have attracted significant interest for their ability to automatically extract features from nonlinear, multi-dimensional material data. These methods have been successfully applied to predict the mechanical properties of composite materials. Table 8 outlines the key advantages and limitations of DL techniques in this context, helping researchers select appropriate methods for their specific applications.

Despite the rapid progress of DIC-ML integration, most existing studies still rely on case-specific training with limited generalization capability. It has been widely reported that supervised ML and DL models trained with inherently limited experimental damage datasets often suffer from poor robustness and weak transferability to unseen conditions [187,288]. Moreover, the majority of reported DIC-based damage identification models are developed and validated under controlled laboratory loading, speckle, and imaging conditions, which significantly restricts their applicability to real-world field environments [289]. In addition, only a limited number of studies explicitly incorporate physical constraints such as strain compatibility, energy balance, or fracture mechanics into the learning process. As highlighted by recent physics-informed ML research, purely data-driven models tend to overfit and show inferior generalization compared to hybrid physics-ML frameworks [288]. These findings reveal a critical gap between current data-driven DIC-ML studies and truly physics-informed damage diagnostics, emphasizing the urgent need for hybrid experimental-mechanics-based DL models.

Overall, AI-based approaches including both ML and DL play an essential role in damage identification for engineering materials. ML methods are effective when working with smaller datasets and offer greater interpretability, while DL excels with large, complex datasets by automating feature extraction and capturing intricate relationships.

Traditional ML methods are generally well suited for smaller datasets, offering reliable accuracy with reduced training times and efficient CPU utilization. However, they often face limitations when dealing with high dimensional data, as careful preprocessing and feature engineering are usually required to achieve satisfactory performance. In contrast, DL techniques demonstrate strong performance with large scale datasets by automatically extracting relevant features without the need for extensive preprocessing. Despite these advantages, DL approaches demand significant computational resources, typically requiring Graphics Processing Unit (GPU) acceleration, and rely on highly complex architectures that can be difficult to interpret.

Although ML and DL techniques show great potential for predicting composite material properties, several key challenges remain in this evolving interdisciplinary field. First, data availability and quality are major concerns. High-quality and different datasets are essential for training accurate AI models. However, their availability is often limited by proprietary restrictions, high costs, or inconsistencies in experimental methodologies. Variations in data formats, measurement techniques, and reporting standards can introduce noise and reduce model reliability. Second, model development and optimization present technical challenges. Designing DL architectures involves complex hyperparameter tuning, with no standard guidelines available. Moreover, many DL models lack interpretability, making it difficult to understand the features driving predictions. Third, generalizability is an ongoing issue. Models trained on specific datasets often struggle to perform well across different material systems, fabrication methods, and environmental conditions. Furthermore, experimental validation remains essential to ensure model reliability, adding time and resource requirements.

To address these challenges, several research opportunities are proposed: Data augmentation using physics-based simulations, GANs, and domain knowledge to enrich training datasets; Hybrid modeling that integrates AI with physic-based approaches to improve accuracy and interpretability; Multi-scale modeling to capture material behavior across micro to macro scales; Explainable AI techniques to increase model transparency and trust; Collaboration with experimentalists for validation and model refinement.

Additionally, the combination of DIC with FEM and the incorporation of ML demonstrate the future direction of fully automated and data-driven DIC systems [290].

Future research on DIC-based ML for damage assessment should focus on three key directions: (i) the development of physics-informed DL models that explicitly incorporate strain compatibility, fracture criteria, and energy-based damage indicators; (ii) the creation of open benchmark DIC datasets with standardized loading conditions to improve model generalization and reproducibility; and (iii) the integration of real-time DIC with lightweight DL architectures for in situ structural health monitoring. These developments are essential to move DIC-ML approaches from laboratory demonstrations toward robust engineering deployment.

## 8. Conclusions

This review examines the integration of DIC with ML techniques for damage identification in engineering materials, highlighting the role of full-field displacement and strain data as physically meaningful indicators of damage initiation and evolution. Compared with conventional vision-based inspection methods that rely primarily on surface appearance, DIC-based approaches provide mechanics-driven information (such as strain localization, displacement discontinuities, and gradient intensification) enabling physics-informed and quantitative damage assessment.

The reviewed literature indicates that conventional ML methods (including SVM, k-NN, Random Forest, and feature-based ANNs) remain effective for small- to medium-sized DIC datasets. Several studies report classification accuracies in the range of 85–95% when physically motivated strain descriptors are carefully designed and evaluated under controlled experimental conditions. These methods offer advantages in interpretability, computational efficiency, and robustness under limited data availability, making them suitable for laboratory-scale studies. However, their performance is strongly dependent on feature selection and preprocessing, and their scalability is limited when applied to high-resolution, full-field DIC strain data.

In contrast, DL-based approaches, particularly CNN-based and fully convolutional architectures (for example, U-Net and DeepLabv3+), demonstrate superior capability in learning complex spatial damage patterns directly from DIC strain fields. Under controlled experimental conditions, these models frequently report damage detection and segmentation accuracies exceeding 95%, with pixel-level crack localization accuracies approaching 98%. Several studies further indicate that DL models can identify damage initiation significantly earlier than visual inspection, by detecting strain localization patterns prior to visible crack formation, while temporal models (for example, RNNs and LSTMs) enable fatigue crack growth prediction with crack-length estimation errors typically below 2–5% in controlled datasets.

Overall, this review highlights that the reliability of ML-assisted DIC is fundamentally governed by DIC data quality, with noise amplification during strain differentiation, sensitivity to speckle quality and lighting conditions, and class imbalance remaining key challenges. Although high accuracies are commonly reported, noticeable performance degradation has been observed under unseen loading conditions or increased noise levels, and uncertainty quantification is still rarely addressed. Nevertheless, the numerical evidence confirms that ML-DIC integration enables earlier, more accurate, and more spatially resolved damage assessment than traditional approaches.

## Figures and Tables

**Table 1:** Imaging-based damage assessment-related methods in mechanical/material engineering.

Category	Techniques	Primary Application	Notes	References
Radiographic	X-ray Radiography, Computed Tomography (CT), Neutron Imaging	Internal defect detection (cracks, porosity, inclusions)	Direct damage detection, widely used for metals, ceramics, composites	[113,114,115]
Ultrasonic	Pulse-Echo, C-Scan, B-Scan, Acoustic Microscopy	Subsurface damages, delamination, bonding evaluation	Direct damage detection, requires coupling medium and signal processing	[116,117]
Infrared (Thermography)	Active Thermography (pulse, lock-in)	Surface and subsurface damages (delamination, voids, disbonds)	Direct damage detection, well-suited for composites and coatings	[118]
Optical/Interferometric	DIC, Digital Volume Correlation (DVC), Speckle, Shearography, Holography	Deformation and strain field measurement, early damage indicators	DIC/DVC mainly for strain analysis (indirect damage detection), Shearography directly for damage localization	[119]

**Table 2:** Summary of selected studies on the use of DIC for damage assessment in engineering materials and structures.

Category	Focus/Definition	Purpose of DIC Use	References	Key Findings
Crack Initiation, Propagation, and Fracture	Detection and tracking of crack initiation, growth, and mixed mode fracture behavior in various materials	To visualize crack development, quantify fracture parameters and validate fracture models	[123,126,127,128,129,130,131,132,133],	DIC effectively captured crack evolution, crack-tip fields, and fatigue behavior, enabled quantitative analysis and model calibration for mixed-mode and inter/intralaminar fractures
General Damage, Strain Irregularity, and Composite Failure	Evaluation of global damage or displacement/strain irregularities in composites and other materials	To measure full-field strain/displacement and identify damaged zones, to improve DIC accuracy under complex conditions	[131,134,135,136,137,138]	Enhanced damage visualization and FE model validation, convolution-based and regularized DIC improved accuracy, DIC successfully combined with hybrid NDT methods
Localized Deformation, Strain Concentration, and Plasticity	Characterization of local strain, large deformation, or yield behavior at macro- to nano-scale	To analyze localized deformation fields, assess strain hardening, and identify interphase or weld zone behavior	[139,140,141,142,143,144,145,146,147,148,149,150,151]	DIC captured large strain and interphase deformation, supported inverse material parameter identification, validated micro- and nano-scale mechanical models
Reviews and Methodological Developments	Review or improvement of DIC algorithms for discontinuities, extreme conditions, or new measurement systems	To summarize DIC applications, identify challenges, and propose methodological or hardware advancements	[20,127,131,138,152]	Provided systematic overviews of DIC capabilities and limitations, proposed algorithmic improvements and optical solutions (e.g., UV/blue DIC for high-temperature use)
Application-Specific Investigations	DIC applied to specific engineering structures or natural materials	To assess structural integrity, thermal deformation, creep, or bio/geological damage mechanisms	[152,153,154,155]	DIC successfully monitored cracking and deformation in civil, thermal, and bio-geological materials, demonstrated potential for SHM and environmental load analysis

**Table 3:** DL methods for damage assessment in engineering materials.

Method	How Damage Is Assessed	Key Advantages	References
CNNs	CNNs automatically learn spatial hierarchies of visual features directly from image data. Networks trained on labeled defect and non-defect images can classify, segment, or localize defects such as cracks, voids, and delamination. Abnormal activation maps or probability outputs highlight defect regions.	Excellent for spatial and texture analysis, requires minimal manual feature extraction, achieves high accuracy for visual inspection tasks.	[235]
RNNs	RNNs capture sequential and temporal dependencies in time-series data. The model learns normal sensor signal patterns and identifies anomalies when observed sequences deviate from predictions.	Effective for real-time monitoring and early fault detection, models dynamic behavior and degradation trends over time.	[236]
AEs	AEs are trained on healthy data to reconstruct inputs. When presented with defective data, the reconstruction error increases and the residual map reveals defect locations. Variational AEs also provide probabilistic anomaly scores.	Fully unsupervised, sensitive to subtle deviations, reconstruction residuals visually indicate defect regions.	[237]
DBNs	DBNs stack Restricted Boltzmann Machines for hierarchical feature learning. After unsupervised pretraining on normal data, the network is fine-tuned to classify defective versus non-defective states based on extracted deep features.	Learns deep nonlinear representations, performs both feature extraction and classification, works effectively with limited labeled data.	[238]
GANs	GANs are composed of a generator and discriminator trained adversarially on normal data. During testing, defective samples yield higher reconstruction errors or low discriminator confidence. GANs are also used for data augmentation by synthesizing realistic defect images.	Highly effective in unsupervised anomaly detection, can generate synthetic defect samples to balance datasets, detects subtle texture irregularities.	[239]
DTL	DTL uses deep networks pre-trained on large image datasets (such as ImageNet) to transfer learned visual features such as edges and textures. These models (VGG, ResNet, EfficientNet) are fine-tuned on small defect datasets, enabling accurate defect classification or segmentation with limited labeled data.	Reduces need for large labeled data, accelerates training, improves generalization to unseen defect types and imaging conditions.	[240]

**Table 4:** Relative improvement (%) of Deep DIC over VIC-2D in displacement and strain measurements for two test cases (values calculated based on the data reported in Ref. [157].).

	Average Displacement		Average Strain		
	*u*	*v*	*ε_xx_*	*ε_yy_*	*ε_xy_*
Test 1	76.9%	86.1%	68.8%	93.0%	48.7%
Test 2	85.5%	81.9%	68.8%	82.1%	58.4%

**Table 5:** Overview of recent studies about composites using ML with DIC for damage identification/detection in composite materials.

Ref.	Type of Anomaly	Type of ML	Specific ML Algorithm	Input to ML	Output from ML	Purpose of Using ML	Purpose of Using DIC
[245]	Damage in CFRP laminates (matrix crack, fiber break, delamination)	Supervised Learning	Semantic Segmentation using DeepLabV3+	DIC-based strain field images	Pixel-wise damage classification	Automate detection and localization of different types of damage in composites	Provide full-field strain images for model input and damage visualization
[186]	Not directly damage-related (supports damage assessment via deformation)	Supervised Learning	StrainNet-LD (CNN with displacement field decomposition)	Image pairs: reference and deformed speckle patterns	Full-field displacement maps	To accurately estimate large displacements beyond traditional DIC limits	Provide high-resolution displacement input under large deformation conditions
[252]	Dynamic Mode I fracture (kink-band propagation)	Supervised Learning	Multilayer Perceptron (MLP) ANN	Strain window, domain height/width (DIC parameters)	Relative importance (RI%) of input parameters	To evaluate sensitivity of fracture toughness to DIC setup parameters	To obtain full-field displacement and strain for J-integral calculation
[256]	Matrix cracking, fiber breakage, delamination	Supervised Learning	SVM	Acoustic Emission (AE) signal features (such as frequency)	Predicted damage type	To classify and monitor damage progression in real-time	To track onset and evolution of damage using AE techniques
[246]	Transverse and longitudinal cracks	Supervised Learning	YOLOv5x (deep object detection)	Sigma distribution maps from DIC	Crack type, segmentation, and location	To perform real-time crack classification and segmentation	To generate strain and Sigma fields sensitive to crack formation

**Table 6:** Overview of recent studies using ML with DIC for damage assessment in metallic materials.

Purpose	ML Approach	Typical Inputs from DIC	ML Output/Purpose	References
Crack detection and localization	Supervised (CNN, U-Net, FNN)	Full-field strain or displacement maps	Crack position, segmentation mask, bounding box	[261,264,266,267,276,278]
Crack propagation and monitoring	Sequential/time-based (RNN, regression)	Strain evolution over cycles, crack length history	Crack growth trend, initiation point prediction	[247,266,273,277]
Anomaly detection	Unsupervised (K-means, clustering)	Maximum shear strain, correlation maps	Crack path segmentation, damage clustering	[247,269,273]
Stress or material property estimation	Supervised/Unsupervised (ANN, Physics-informed CNN)	2D/3D displacement fields	Internal stress field, material inhomogeneity mapping	[269,270,274]
Physics-informed ML/Synthetic data generation	PG-cGAN, PG-GAN, mechanics-informed CNN	Displacement or strain with physics constraints	Realistic or physics-consistent strain/displacement fields	[272,279]
Support for numerical modeling/inverse analysis	Physics-based ML + DIC	Boundary input displacement fields	Data generation for model calibration or FEM updates	[272,279]

**Table 7:** Traditional ML techniques versus their advantages and disadvantages.

ML Technique	Advantage	Disadvantage
SVM	Provides high accuracy and speed for smaller datasets, handles high-dimensional data effectively, memory usage is relatively efficient.	Not efficient for very large datasets, struggles with noisy data.
K-NN	Simple structure, easy to implement, robust to noisy data, supported by a well-established theoretical foundation.	Slow when dealing with large datasets, computationally demanding, poor results with high-dimensional data, requires considerable storage, sensitive to the choice of k.
Decision Tree	Easy to interpret and explain, results can be visualized clearly.	Easily prone to overfitting, training can take longer, may require additional domain knowledge.
Random Forest	Simple to understand, computationally inexpensive, performs well on high-dimensional datasets.	Can still be prone to overfitting.
ANN	Capable of parallel data processing, high prediction accuracy and fast performance, effective at modeling complex nonlinear relationships, works well with larger datasets.	Requires heavy computation, risk of overfitting on small datasets, often criticized for being a ‘black box’ with low transparency.

**Table 8:** Advantages and disadvantages of DL and traditional ML methods.

DL Method	Advantages	Disadvantages
CNN	Well suited for analyzing multi-dimensional data, especially images. Very effective at feature extraction and strong in local feature detection.	Complex design that requires long training time. Needs large training datasets and is prone to overfitting.
RNN	Good for sequential and time-series data. Can effectively capture temporal changes and patterns.	Training and implementation are challenging due to their complex structure.
AE	Simple to implement and computationally efficient. Capable of learning rich and compressed representations.	Needs large datasets for training. Performance decreases when noise dominates or when errors appear in the early layers.
DBN	Effective with complex data, even without extensive preprocessing. Can extract high-level features and reduce dependency on labeled data.	Training is slow and computationally heavy due to complex initialization. Inference with multiple hidden layers can be difficult.
GAN	Powerful for generating synthetic data from limited training samples.	Difficult to optimize. Data generation is limited when training data are scarce.

## Data Availability

No new data were created or analyzed in this study. Data sharing is not applicable to this article.
